# Homocysteine concentration and adenosine A_2A_ receptor production by peripheral blood mononuclear cells in coronary artery disease patients

**DOI:** 10.1111/jcmm.15527

**Published:** 2020-06-29

**Authors:** Pierre Deharo, Marion Marlinge, Clair Guiol, Donato Vairo, Julien Fromonot, Patrick Mace, Mohamed Chefrour, Marguerite Gastaldi, Laurie Bruzzese, Melanie Gaubert, Marine Gaudry, Nathalie Kipson, Christine Criado, Thomas Cuisset, Franck Paganelli, Jean Ruf, Regis Guieu, Emmanuel Fenouillet, Giovanna Mottola

**Affiliations:** ^1^ C2VN INSERM INRA Aix Marseille University Marseille France; ^2^ Laboratory of Biochemistry Timone University Hospital Marseille France; ^3^ Department of Vascular Surgery Timone University Hospital Marseille France; ^4^ Department of Cardiology Hospital Nord Marseille and C2VN Marseille France; ^5^ Department of Cardiology Timone University Hospital Marseille France; ^6^ CNRS Institut des Sciences Biologiques Paris France

**Keywords:** A_2A_ receptor, adenosine, coronary artery disease, homocysteine

## Abstract

Hyperhomocysteinemia is associated with coronary artery disease (CAD). The mechanistic aspects of this relationship are unclear. In CAD patients, homocysteine (HCy) concentration correlates with plasma level of adenosine that controls the coronary circulation via the activation of adenosine A_2A_ receptors (A_2A_R). We addressed in CAD patients the relationship between HCy and A_2A_R production, and in cellulo the effect of HCy on A_2A_R function. 46 patients with CAD and 20 control healthy subjects were included. We evaluated A_2A_R production by peripheral blood mononuclear cells using Western blotting. We studied in cellulo (CEM human T cells) the effect of HCy on A_2A_ R production as well as on basal and stimulated cAMP production following A_2A_ R activation by an agonist‐like monoclonal antibody. HCy concentration was higher in CAD patients vs controls (median, range: 16.6 [7‐45] vs 8 [5‐12] µM, *P* < 0.001). A_2A_ R production was lower in patients vs controls (1.1[0.62‐1.6] vs 1.53[0.7‐1.9] arbitrary units, *P* < 0.001). We observed a negative correlation between HCy concentration and A_2A_ R production (*r* = −0.43; *P* < 0.0001), with decreased A_2A_ R production above 25 µM HCy. In cellulo, HCy inhibited A_2A_R production, as well as basal and stimulated cAMP production. In conclusion, HCy is negatively associated with A_2A_ R production in CAD patients, as well as with A_2A_ R and cAMP production in cellulo. The decrease in A_2A_ R production and function, which is known to hamper coronary blood flow and promote inflammation, may support CAD pathogenesis.

## 1 INTRODUCTION

Homocysteine (HCy) is a thiol‐containing amino acid intermediate the metabolism of which is linked to those of methionine, uric acid and adenosine. HCy and adenosine concentrations are correlated at least in coronary artery disease (CAD) patients.^1^ Adenosine is an ATP derivative that is released by endothelial cells and myocytes during ischaemia, hypoxia or inflammation.^2^, ^3^ Adenosine impacts the cardiovascular system via the activation of its receptors namely A_1_ R, A_2A_ R, A_2B_ R or A_3_ R pending on their pharmacological properties.^4^ A_2A_ R activation exerts artery vasodilation via cAMP production^5^ and specially coronary vasodilation,^6^ cAMP production and coronary vasodilation being correlated.^7^

Chronic ischaemia elicits coronary vasodilation in the myocardium.^8^ This adaptive response is partly due to adenosine release that improves coronary blood flow via activation of A_2A_ R^6^, ^9^, ^10^ and A_2B_ R.^9^, ^10^ Although acute release of adenosine leads to coronary vasodilation and may be consequently beneficial for the myocardium, chronic exposure to high adenosine level may have deleterious effects.^11^

Hyperhomocysteinemia (HHCy) is associated with cardiovascular disease^12^, ^13^ and independently associated with CAD^14^, ^15^ and myocardial infarction‐induced death.^16^ It was also found that HHCy is correlated with CAD severity^1^, ^17^ although the mechanistic aspects of this relationship are unclear. Finally, HHCy is associated with increased oxidative stress^18^ and endothelial dysfunction.^19^ However the precise mechanism by which HHCy participates into CAD progression remains controversial because homocysteine‐lowering therapy does not affect the inflammatory status of CAD patients^20^ and poorly influences cardiovascular risk.^21^

Peripheral blood mononuclear cells (PBMC) can be easily sampled and the behaviour of adenosine receptors produced on PBMC mirrors their counterparts in heart,^22^ and in coronary arteries.^23^ Therefore, PBMCs are useful to address A_2A_ R pharmacological properties in patients and to evaluate the influence of HCy on adenosine receptors of the vascular system. We previously observed a decrease in A_2A_ R production by PBMC of CAD patients^24^ the cause of which not yet investigated.

Thus, we evaluated here the relationship between HCy concentration and A_2A_ R production in CAD patients. We also examined in cellulo the effect of HCy concentration on A_2A_ R and cAMP production in basal conditions and after agonist exposure.

## 2 MATERIALS AND METHODS

### 2.1 Patients

#### 2.1.1 Panel size

A difference in APL or A_2A_ R expression >20%‐25% was considered to have pathophysiological relevance; accordingly, a panel of 30‐40 subjects was considered to be sufficient to provide statistical signification.

#### 2.1.2 Study population

We recruited 46 patients (13 women and 33 men, mean age 69.3 ± 11.6 years) admitted for coronary angiography in the department of Cardiology, University Hospital, Marseille, between January 2016 and January 2018. Clinical presentation could be an acute coronary syndrome or stable angina. The patients included presented with a significant CAD defined by an angiographic stenosis ≥50%. Exclusion criteria for the study were creatinine clearance <25 mL/min and age <18 or >80 years. Twenty healthy subjects (7 women and 13 men, mean age 63 ± 7) from the medical staff, matched for age and sex, were used as controls for the adenosinergic profile. They were without history of cardiovascular or inflammatory disease and not under any medical treatment. The ethic committee of our institution (CPP number: 17 05 45; protocol number: 2017‐36) approved the study protocol, and patients and healthy subjects gave written informed consent for participation.

### 2.2 Blood sample collection

Blood sample collection was performed before invasive coronary angiography (ICA). For adenosine measurement, blood (3 mL) was collected from a cubital vein using a syringe containing 2 mL of a cold stop solution to prevent uptake and degradation of adenosine by red blood cells.^25^, ^26^ The stop solution was composed of dipyridamole (0.2 mM), ethylene diamine tetracetic acid disodium (4 mM), erythro −9‐(2‐hydroxy‐3‐nonyl adenine (5 µM) l, ‐methyleneadenosine 5′ diphosphate (79 µM), coformycine 10 μg/mL, and heparin sulphate 1 IU/mL (Sigma Aldrich, St Quentin Fallavier, France). After collection, samples were placed on ice then centrifuged (4°C, 1500 *g*).

### 2.3 Adenosine concentration measurement

Adenosine plasma concentration (APC) as well as adenosine measurement in free cell culture supernatant were performed as previously described by liquid chromatography‐tandem mass spectrometry after extraction^26^, ^27^ using a Shimadzu UFLC XR system (Shimadzu, Marne la Vallée, France).

Plasma extraction procedure: internal standard solution with 2‐Chloro adenosine was prepared (300 nM in water). Plasma sample (100 µL) was transferred into a microfuge tube. Each sample was spiked with internal standard solution (50 µL) and methanol (300 µL) and vortexed for 1 minute. Samples were then centrifuged (4°C, 10 minutes, 13 300 *g*). Supernatant was then evaporated to dryness at 60°C under nitrogen. Formic acid (0.1% in water; 150 µL) was then added and quickly vortexed prior to transfer in an HPLC auto‐sampler vial. The intra‐assay coefficient of variation (CV) was <10%.

### 2.4 HCy measurement

Blood was collected in a tube with EDTA and centrifuged (4°C, 10 minutes, 2000 *g*). Plasma was frozen and stored until assay. Total homocysteine was quantified with the LC‐MS Clinmass® ‘Homocysteine in plasma/serum’ kit (Recipe, Germany). Supernatants were analysed using a Shimadzu UFLC XR system consisting of two LC‐20ADXR binary pumps, a DGU20A5R vacuum degasser, a CT0‐20AC thermostated column oven and a SIL‐20ACXR cooled auto sampler (Shimadzu). The LC system was interfaced with an ABSciex 4500 triple quadrupole mass spectrometer (Les Ulis, France) operating with an electrospray ionization source (ESI) using nitrogen (purity: 99.99%). The intra‐ or inter‐assay CV was <5%.

### 2.5 PBMC A_2A_R production

The procedure has been described.^28^, ^29^, ^30^ In brief, blood was collected in a tube containing sodium citrate, a polyester gel and a density gradient liquid (Vacutainer CPT, Beckton Dickinson) prior to centrifugation (20 minutes; 1700 *g* at room temperature). The PBMC layer was collected and washed twice using phosphate‐buffered saline prior to treatment with lysis buffer and sonication. Samples (0.25 × 10^6^ cells) were then submitted to standard 12% polyacrylamide gel electrophoresis under reducing conditions prior to transfer onto a PVDF membrane. The filter was then incubated with Adonis (1 μg/mL), a homemade IgM, kappa mouse monoclonal antibody directed against a linear epitope of A_2A_R (By et al 2009). Detection was performed using phosphatase alkaline‐labelled anti‐mouse antibodies and phosphatase alkaline substrate. The 45‐kDa band corresponding to A_2A_R was submitted to densitometry analysis using the ImageJ 1.42q software (National Institutes of Health) and results were expressed as arbitrary units (AU) as previously described^31^, ^32^ (the ratio of pixels generated by the A_2A_R band to pixels generated by the background signal was calculated). In these conditions, the intra‐ or inter‐assay CV was <10%.

### 2.6 Cell culture experiments

Human lymphoblastoid T cells (CEM cells; ATCC CCL 119) that express A_2A_ R were cultured in RPMI 1640 medium in the presence of bovine foetal serum (10%) and C02 (5%) at 37°C. CEM cells (5 × 10^5^/mL) were then seeded in flasks (75‐cm^2^, 50 mL) and cultured for 48 hours in the presence of HCy (50 and 200 µM). HCy concentration was readjusted every 4 hours according to HCy dosage (see above). Duplicates were performed. Cell viability was addressed using the Trypan Blue dye exclusion method.

### 2.7 Adenosine concentration measurement in cell culture medium

Cell‐free supernatant was blotted on a Whatmann blot paper (6 mm diam.) prior to extraction using a mixture of methanol (400 µL) and internal standard (see above) for 90 minutes at 45°C. After extraction, an aliquot (350 μL) was evaporated to dryness at 60°C under nitrogen. Formic acid (0.1% in water; 150 µL) was added and vortexed prior to transfer into an HPLC auto‐sampler vial. Dosage was then performed using LC‐MS/MS as described above.

### 2.8 Adenosine deaminase activity (ADA) measurement

Ado (28 mM; 750 μL) was mixed with cell culture medium (750 μL) in NaCl 0.9% (2 mL final volume). Aliquots were then incubated (40 minutes, 37°C). The reaction was started by adding the substrate and was stopped by cold immersion. COBAS 8000 apparatus (Roche®, Geneva, Switzerland) was used to quantify ammonia concentration. The intra‐ and inter‐assay coefficients of variation ranged between 3% and 5%.

### 2.9 cAMP dosage

The method has been previously described.^25^ PBMC (2 × 10^5^ per well) were incubated with increasing concentrations of HCy during 48 hours. IBMX (1 mM; inhibitor of phosphodiesterase to prevent cAMP degradation) and Adonis (1 µM; used as an agonist) were then added for 90 minutes. Dodecyltrimethylammonium bromide acetate buffer was used to stop the incubation step. Dosage of cAMP was performed in duplicate using the Amersham Biotrak Kit (distributed by Sigma Aldrich, St Quentin Fallavier, France). The intra or inter‐assay CV was <10%.

### 2.10 Statistical analysis

Data were described by mean and standard deviation or median and interquartile range. Correlations between biological parameters were quantified and tested using Pearson's correlation coefficient. Comparisons of biological parameters between patients and controls were performed using a variance analysis (ANOVA two ways). All statistical tests were two‐sided and *P* values less than 0.05 were considered statistically significant. Analysis were performed using the SPSS software (version 13.0 2004; SPSS Inc, Chicago, IL, USA).

## 3 RESULTS

### 3.1 Patients

Clinical characteristics of the 46 patients are summarized in Table 1; treatments are reported in Table 2. Of note, 13 (28%) women had a mean age of 69.3 ± 11.6 years and mean BMI of 26.6 ± 4.3 kg/m^2^ and 14 (30%) patients presented with ACS.

**Table 1:** Clinical characteristics

Characteristics	All patients (n = 46)
Female (n, %)	13 (28%)
Age (years; M ± SD)	69.3 ± 11.6
BMI (kg/m^2^; M ± SD)	26.6 ± 4.3
Hypertension (n, %)	33 (72%)
Type II diabetes (n, %)	18 (39%)
Dyslipidaemia (n, %)	25 (54%)
Current smoker (n, %)	12 (26%)
Previous CAD (n, %)	9 (20%)
ACS (n, %)	14 (30%)
One‐vessel disease (n, %)	19 (41%)
Two‐vessel disease (n, %)	10 (22%)
Three‐vessel disease (n, %)	17 (37%)
PCI (n, %)	34 (74%)
CRP (mg/L; M ± SD)	4.2 ± 2.5
Fibrinogen (g/L; M ± SD)	3.9 ± 1.0
Triglyceride (g/L; M ± SD)	1.9 ± 1.4
Cholesterol (g/L; M ± SD)	1.7 ± 0.5
HDL (g/L; M ± SD)	0.4 ± 0.1
LDL (g/L; M ± SD)	0.9 ± 0.4
Glycaemia (mmol/L; M ± SD)	7.1 ± 2.7
Creatinine (µmol/L; M ± SD)	94.3 ± 25.6
HbA1c (%; M ± SD)	6.8 ± 1.3
Haemoglobin (g/dL; M ± SD)	13.5 ± 1.8
Platelets (G/L; M ± SD)	256 ± 61
Lymphocytes (G/L: M ± SD)	1.989 ± 950*

Abbreviations: n: number; M: mean; SD: standard deviation; BMI: Body Mass Index; CAD: coronary artery disease; RAS: Renin Angiotensin System; ACS: Acute Coronary Syndrome; PCI: percutaneous coronary intervention; CRP: C‐reactive protein; HDL: High density lipoprotein; LDL: low density lipoprotein; HbA1C: glycosylated haemoglobin.

* Normal range 1.000‐4.000.

**Table 2:** Treatment of CAD patients

Betablocker	n = 27 (59%)
Bisoprolol	12 (26)
Metoprolol	11 (24)
Propranolol	4 (9)
ACE inhibitors	5 (11)
Ramipril	5 (11)
RAS inhibitors	28 (61)
Valsartan	18 (39)
Irbesartan	6 (13)
Candesartan	4 (9)
Calcium blocker	6 (13)
Nicardipine	3 (7)
Amlodipine	1 (2)
Lercanidipine	1 (2)
Diltiazem	1 (2)
Fibrates	3 (6)
Fenofibrate	3 (6)
Statin	34 (73)
Atorvastatin	14 (30)
Pravastatin	8 (17)
Simvastatin	8 (17)
Rosuvastatin	4 (9)
Aspirin	36 (78)
P2Y12 inhibitor	18 (39)
Clopidogrel	18 (39)
Diuretics	6 (13)
Furosemide	6 (13)
Anti‐diabetics	14 (30)
Metformin	10 (21)
Gliclazide	4 (9)

**Figure 1:**
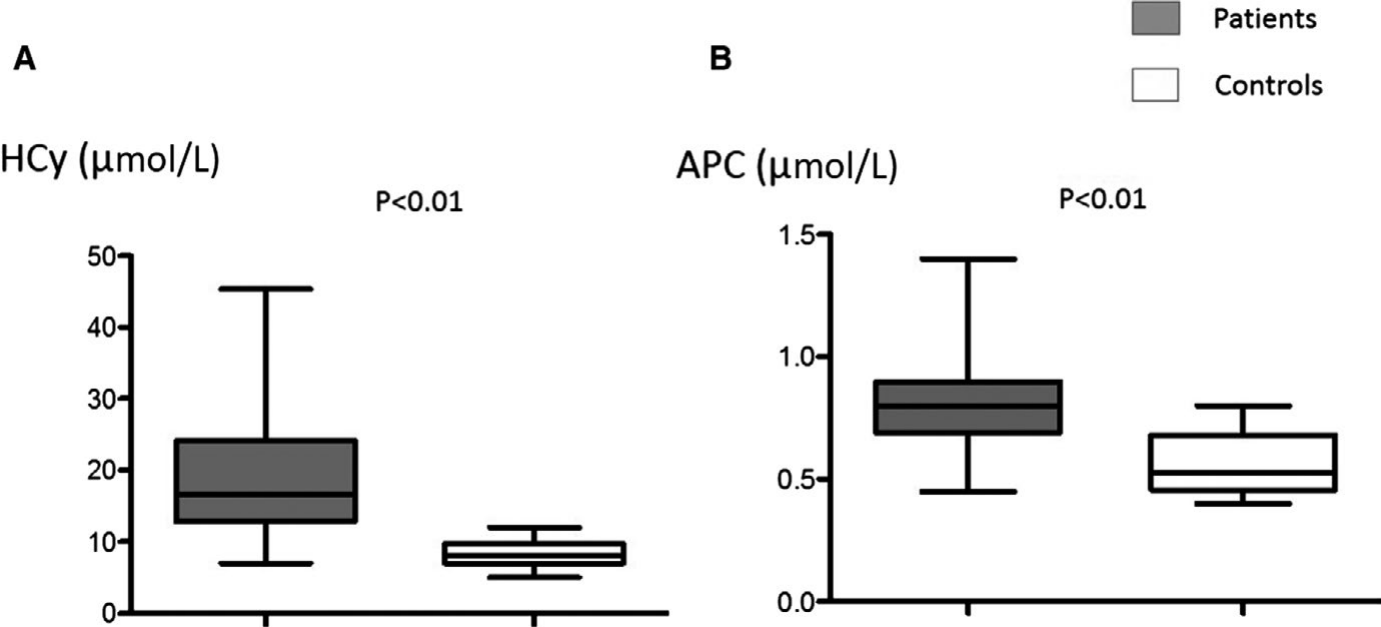
Homocysteine concentrations (HCy, A) and adenosine plasma concentrations (APC, B) in coronary artery disease patients (n = 46) and healthy subjects (controls; n = 20)

**Figure 2:**
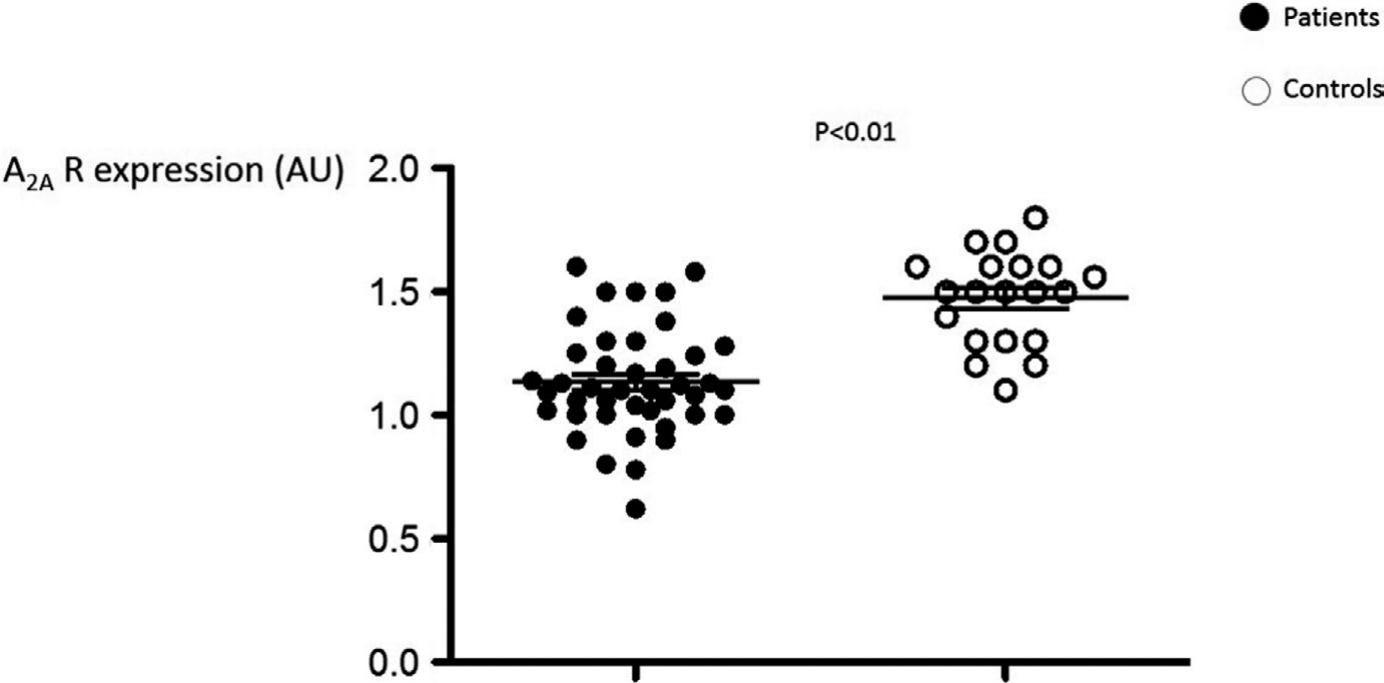
Adenosine A_2A_ receptor (A_2A_ R) production by peripheral blood mononuclear cells from coronary artery disease patients (n = 46) and healthy subjects (controls, n = 20). A_2A_ R production was evaluated by Western blotting and expressed as arbitrary units (AU)

HCy and adenosine plasma concentrations (APC) were higher in patients vs controls (median, range: 16.6 [7‐45] vs 8 [5‐12] µM, *P* < 0.001, Figure 1A; 0.8 [0.45‐1.4] vs 0.52 [0.4‐0.8] µM, *P* < 0.01, Figure 1B, respectively). A_2A_ R expression was lower in patients vs controls (1.1 [0.62‐1.6] vs 1.53 [1.1‐1.7] AU, *P* < 0.001, Figure 2). It is of note that overall the presence of diabetes, HTA, dyslipidaemia or ACS did not significantly modify HCy, A_2A_ R production or APC (Table S1). No significant difference was found concerning gender in patients (HCy: men vs women: 18.7 ± 9.4 vs 16.9 ± 5 µM, *P* = 0.9; APC: 0.78 ± 0.22 vs 0.76 ± 0.14 µM, *P* = 0.9; A_2A_ R production: 1.08 ± 0.25 vs 1.12 ± 0.19 Arbitrary Units, *P* = .39).

**Figure 3:**
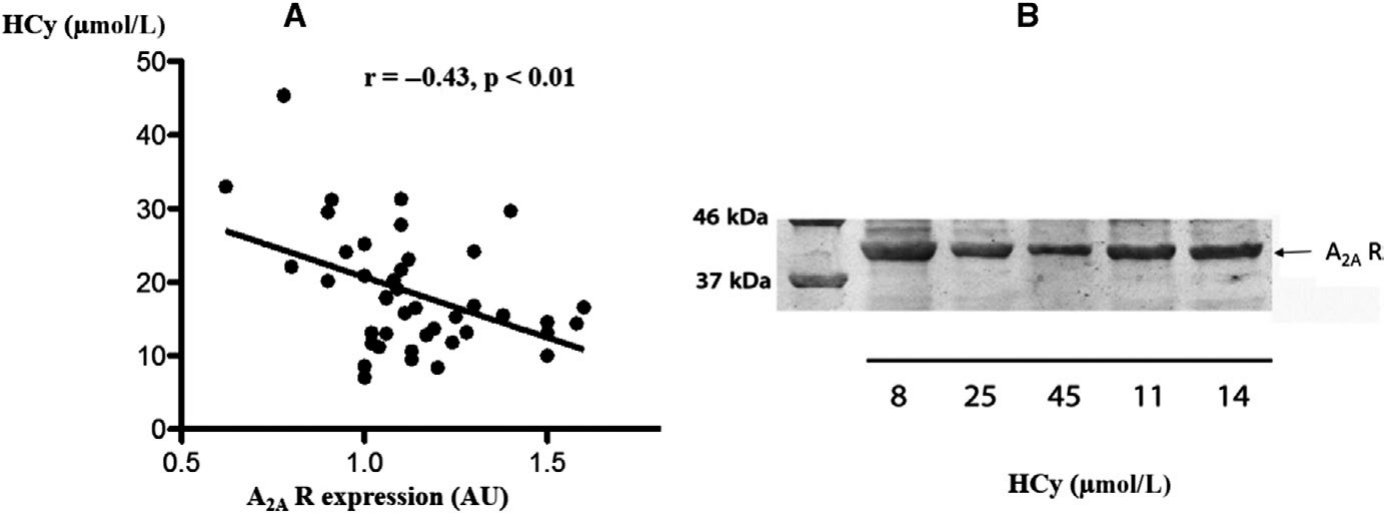
A, Correlation curve (Pearson) between plasma homocysteine concentration (HCy) and A_2A_ receptor (A_2A_ R) production by peripheral blood mononuclear cells in 46 coronary artery disease patients. B, A_2A_R production (Western blot; apparent MW of the molecular species: 45 kDa) and HCy concentration found in selected patients

In patients, we observed a negative correlation between HCy concentration and A_2A_ R production (*r* = −0.43; *P* < 0.0001; Figure 3A), with decreased A_2A_ R production above 25 µM HCy (Figure 3B). We did not find a correlation between C reactive protein and A_2A_R production (*r* = 0.16, *P* = 0.41) while a trend in correlation was found between HCy and C reactive protein concentrations (*r* = 0.33, *P* = 0.06).

### 3.2 In cellulo study

Using CEM cells, we observed that HCy inhibited in a concentration‐ and time‐dependent manner (a) A_2A_ R production (−39% and −46% at 50 µM at 24 and 48 hours, respectively; −54% and −69% at 200 µM at 24 and 48 hours, respectively; Figure S1) and (b) cAMP production (basal conditions: −24%, −55% and −69% at 25, 50 and 200 µM, respectively; Figure S2A). We also observed that while Adonis (0.9 µM) increased cAMP production (+153% vs basal condition), HCy inhibited the Adonis‐induced cAMP production increase in a concentration dependent manner (−18%, −66% and −75% at 25, 50 and 200 µM, respectively; Figure S2B). It is of note that (a) the adenosine concentration level was very weak in culture medium (<0.025 µM) irrespective of the experimental condition; (b) ADA per cell did not increase significantly as a function of time or HCy concentration (Table S2); (c) HCy (25‐200 µM) did not influence cell proliferation.

## 4 DISCUSSION

We report here a negative correlation between HCy concentration and A_2A_ R production by PBMC in CAD patients. We also found that HCy concentrations in the range of those measured in CAD patients decreased both A_2A_ R and cAMP production in CEM human T cells. In the latter conditions, it is likely that down regulation of A_2A_ R resulted from HCy and not from changes in adenosine concentration or ADA.

These results support and expand our previous observation that took advantage of an in cellulo model of inflammation and hypoxia, which may be considered as reflecting CAD conditions. Using CEM T cells, we previously showed that HCy decreases A_2A_ R and cAMP production via H_2_S and NF kappa B pathway.^33^, ^34^ In patients, low A_2A_ R and cAMP production may participate in CAD in three ways: (a) by altering the adaptive vasodilation of coronary arteries when oxygen supply is needed, an hypothesis that is supported by the correlation found between the CAD gravity score (Syntax score) and HCy concentration^1^; (b) by inhibiting the adenosinergic T cell immunosuppression mechanism via H_2_S production that, in turn, promotes inflammation^33^; and (c) by favouring platelet aggregation and activation through HCy/H_2_S pathway, thus contributing to atherothrombosis, stroke or myocardial infarction.^35^

It was reported that an increase in 5 µM HCy promotes the incidence of CAD^36^ and that HHCy is associated with restenosis of CAD patients treated by percutaneous intervention^37^ as well as with cardiovascular causes of death.^38^ Furthermore, an association between CRP and HCy concentrations was reported in patients with acute myocardial infarction.^39^ The effects of HCy in CAD may result from an increase in oxidative stress in the vascular endothelium.^40^

It was shown that HCy promotes endothelial cell dysfunction via the up‐regulation of p66shc expression following hypomethylation of the promoter.^41^ Additionally, HCy promotes vascular inflammation and atherosclerosis through hypermethylation of the *SMAD7* promoter.^42^ A mild elevation of HCy, associated with a specific polymorphism of the enzyme methyl tetrahydrofolates (MTHF) was also suggested to modify MTHF activity.^43^ In addition, HCy was shown to interfere with cell viability,^44^ migration^45^, ^46^ and cytokine production^45^, ^47^, ^48^ in various cell populations although these effects have been found for homocysteine concentrations that are only very rarely found in CAD patients (above 300 µM).

With regard to the adenosinergic system, we observed high APC in patients vs controls. High APC has been previously described in CAD,^1^, ^23^, ^24^ adenosine being released by endothelial and muscle cells to control inflammation,^3^, ^49^ ischaemia^2^, ^50^ and hypoxia.^2^, ^49^ However, part of adenosine production may be secondary to high HCy level inasmuch as HHCy induces a rapid metabolism of ATP, ADP and AMP into adenosine via macrophage ectonucleotidases.^50^ Activation of A_2A_R has anti‐inflammatory effects.^51^, ^52^ Conversely, the decrease in A_2A_R activation found in CAD patients probably promotes inflammation that in turn promotes atherosclerosis.^53^, ^54^

In summary, we found that HCy concentrations measured in CAD patients are associated with low production of A_2A_R in CAD patients as well as with low production of cAMP in cellulo. These data are consistent with the possibility that HCy participates in CAD pathophysiology by reducing coronary blood flow and by promoting inflammation and atherosclerosis.

### 4.1 Limitations

The possible influence of A_1_ and/or A_2_B receptors on the effects of HCy on cAMP production was not addressed in this study.

## 5 CONCLUSION

HCy is negatively correlated with A_2A_ R production in CAD patients, and negatively associated with A_2A_ R production and cAMP level in cellulo. The decrease in A_2A_ R production and cAMP level, which is known to hamper coronary blood flow and promote inflammation, may support CAD pathogenesis.

## CONFLICT OF INTEREST

The authors have nothing to disclose.

## AUTHOR CONTRIBUTION

**Pierre Deharo:** Conceptualization (lead); Resources (equal); Software (equal). **Marion Marlinge:** Data curation (equal). **Clair Guiol:** Supervision (equal). **Donato Vairo:** Investigation (equal). **Julien Fromonot:** Software (equal). **Patrick Mace:** Investigation (equal). **Mohamed Chefrour:** Visualization (equal). **Marguerite Gastaldi:** Project administration (equal). **Laurie Bruzzese:** Validation (equal). **Melanie Gaubert:** Project administration (equal). **Marine Gaudry:** Investigation (equal). **Nathalie Kipson:** Resources (equal). **Christine Criado:** Formal analysis (equal). **Thomas Cuisset:** Resources (equal). **Franck Paganelli:** Data curation (equal). **Jean Ruf:** Writing‐original draft (equal). **Regis Guieu:** Writing‐review & editing (equal). **Emmanuel Fenouillet:** Validation (equal). **Giovanna Mottola:** Conceptualization (equal); Visualization (equal).

## Supporting information

Fig S1

Fig S2

Table S1

Table S2

## Data Availability

Non available.
