# Supplementary figures and images for: Homocysteine concentration and adenosine A2A receptor production by peripheral blood mononuclear cells in coronary artery disease patients
Source: J Cell Mol Med. 2020 Jun 29;24(16):8942–9. doi: 10.1111/jcmm.15527 (PMC7417719; doi:10.1111/jcmm.15527)

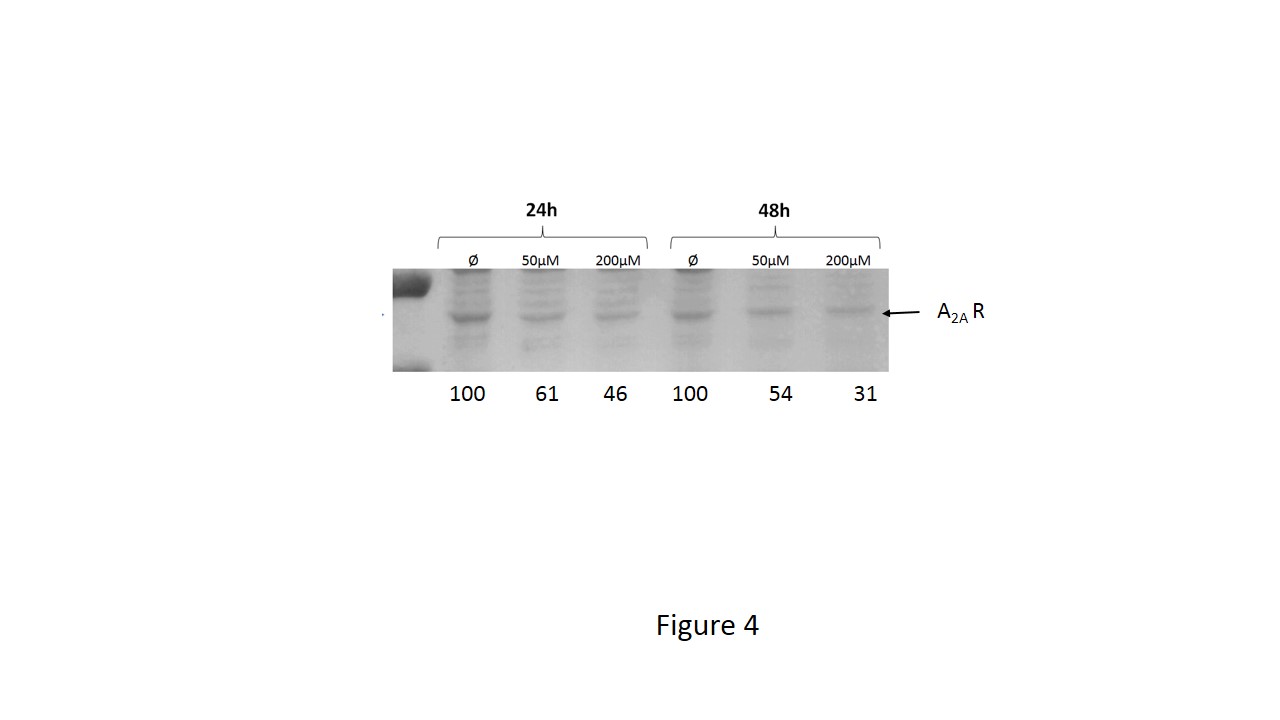

Supplement: Supplementary file 1 — Fig S1 [file JCMM-24-8942-s001.tiff]

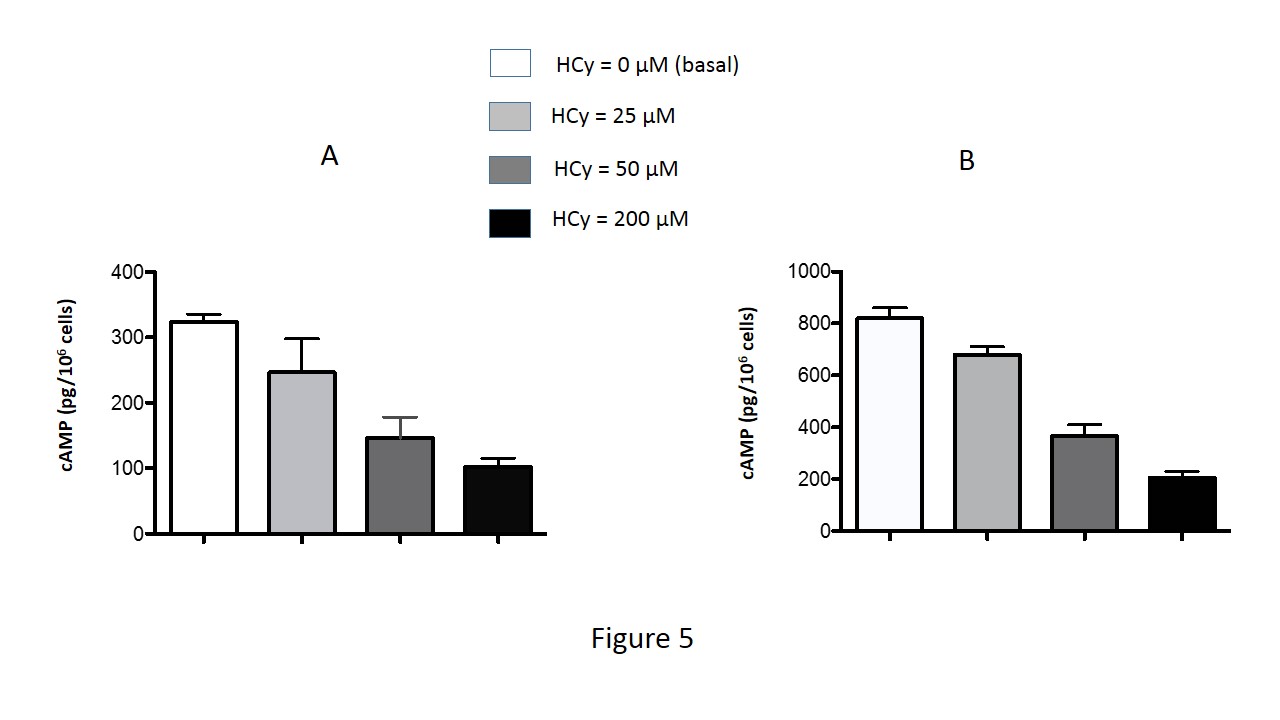

Supplement: Supplementary file 2 — Fig S2 [file JCMM-24-8942-s002.tiff]
