# Supplementary material for: Homocysteine concentration and adenosine A2A receptor production by peripheral blood mononuclear cells in coronary artery disease patients
Source: J Cell Mol Med. 2020 Jun 29;24(16):8942–9. doi: 10.1111/jcmm.15527 (PMC7417719; doi:10.1111/jcmm.15527)
Supplement: Supplementary file 3 — Table S1 [file JCMM-24-8942-s003.docx]

| **Mean**  **±SD** | **HTA vs non HTA** | **Dyslipidemia vs non Dyslipidemia** | **Diabetus vs non diabetus** | **ACS vs non ACS** |
| --- | --- | --- | --- | --- |
| **HcY**  **(µM)** | 18±8 vs 20.6±7.7 ; p=0.26 | 16.8±7.9 vs 20.5±8.5 ; p=0.12 | 17.8±7.9 vs 19.38±7.9 ; p=0.52 | 19.5±9 vs 19.1±7.8 ; p=0.7 |
| **A2A R (AU)** | 1.13±0.18 vs 1 .12±0.3 ;p=0.7 | 1.11 ±0.2 vs 1.14±0.22 ; p=0.8 | 1.09±0.21 vs 1.13±0.3 ; p= 0.49 | 1.12±0.17 vs 1.14±0.2 ; p=0.8 |
| **APC**  **(µM)** | 0.78±0.2 vs 0.78±0.16 ; p=0.8 | 0.74±0.17 vs 0.8±0.2, p= 0.56 | 0.78±0.25 vs0.71±0.24 ; p=0.89 | 0.98±0.19 vs 0.69±0.12 ; p<0.01 |

Supplemental table 1: Impact of associated pathology homocysteine(HCy) or adenosine (APC) plasma concentrations and on adenosine A_2A_ receptor production, evaluated on peripheral blood mononulcear cells (PBMC).

AU : arbitrary units. ACS : Acute cornary syndrome
