# Supplementary material for: Homocysteine concentration and adenosine A2A receptor production by peripheral blood mononuclear cells in coronary artery disease patients
Source: J Cell Mol Med. 2020 Jun 29;24(16):8942–9. doi: 10.1111/jcmm.15527 (PMC7417719; doi:10.1111/jcmm.15527)
Supplement: Supplementary file 4 — Table S2 [file JCMM-24-8942-s004.docx]

ADA (IU/10^6^ cells)

| HCy concentration  (µM) | 0 | 24 hrs | 48 hrs |
| --- | --- | --- | --- |
| 0 | 19 | 26 | 27 |
| 25 | 19 | 26 | 19 |
| 50 | 19 | 17 | 22 |
| 200 | 19 | 21 | 17 |

Supplemental table 2 : Adenosine deaminase activity (ADA) as a

function of time and HCy concentration In CEM cell culture.
